# Syndrome de Peutz-Jeghers, à propos de 3 cas dans la fratrie

**Published:** 2012-03-27

**Authors:** Kaoutar Zinelabidine, Meriame Meziane, Fatima Zahra Mernissi

**Affiliations:** 1 Service Dermatologie (E4), Centre Hospitalo-Universitaire Hassan II, Fès, Maroc

**Keywords:** Peutz-Jeghers, lentiginose, pigmentation cutanéo-muqueuse, Maroc

## Abstract

Le SPJ est une maladie rare de transmission autosomique dominante, défini par l’association d’une atteinte cutanée à type de lentiginose péri-orificielle d’une atteinte digestive, pulmonaire et des organes reproducteurs. Le gène de PJS a été localisé sur le chromosome 19p13.13. Les signes cutanés sont affichants et constituent un signe révélateur de la maladie, ils sont à type de lentigines, Ils siègent le plus souvent sur les lèvres, la muqueuse buccale. Les polypes digestifs constituent le deuxième signe cardinal de ce syndrome et ils peuvent révéler la maladie lorsqu’ils se manifestent d’emblée par leur complication telles les hémorragies digestives et les obstructions. Les personnes atteintes de SPJ courent un risque accru de cancers, le risque cumulatif de cancer est estimé à 93%, Les organes les plus fréquemment touchés sont le tractus gastro-intestinal (oesophage, estomac, intestin grêle, côlon, rectum et pancréas), le poumon, la prostate, le sein et les organes reproducteurs.

La prise en charge des SPJ est basée principalement sur la surveillance et le traitement des polypes hamartomateux. Il n′existe aucun traitement standarisé pour la pigmentation cutanéo-muqueuse. Le traitement fait appel à la cryochirurgie, la dermabrasion et au laser Q-switched.

## Introduction

Le syndrome de Peutz-Jeghers (SPJ) est une affection autosomique dominante rare, associant une lentiginose surtout péri orificielle à des hamartomes digestifs, et comportant un risque d’association à des cancers. Nous rapportant 3 cas dans la fratrie avec revue de la littérature.

## Patient et observation

**Figure 1:**
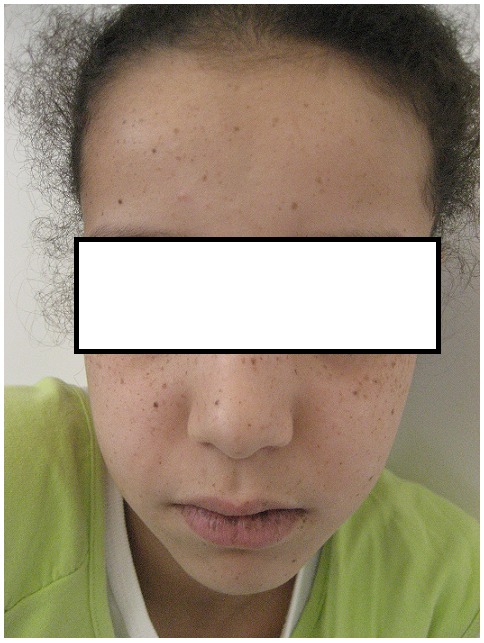
Lentigines au niveau du dos du nez, des 2 joues et sur les 2 lèvres

**Figure 2:**
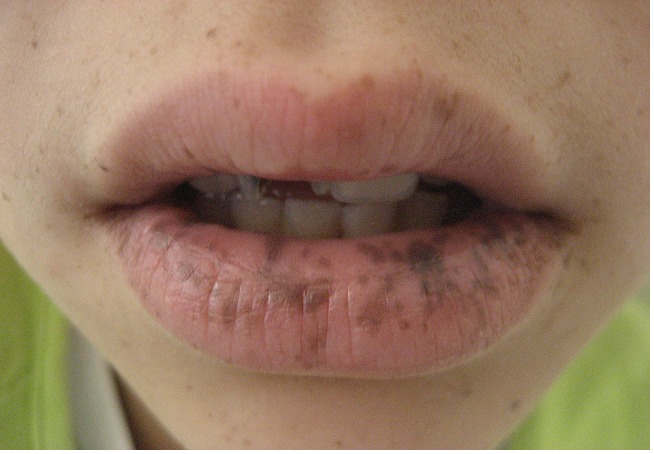
Lentigines au niveau des 2 lèvres

**Figure 3:**
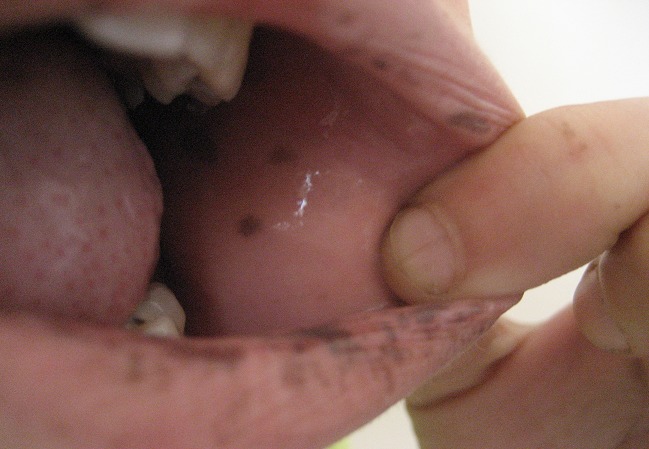
Lentigines au niveau de la muqueuse buccale

Une jeune fille N.K âgée de 13 ans, sans notion de consanguinité, sans antécédents pathologiques notables, présentait depuis l’âge de 5 ans des lésions hyperpigmentés au niveau des 2 joues, l’évolution a été marquée par l’extension de ces lésions brunâtres. L’examen à l’admission objectivait des lentigines au niveau du dos du nez, des 2 joues, sur les 2 lèvres, sur la muqueuse buccale, au niveau palmo-plantaire (Figure 1, Figure 2, Figure 3). L’examen des autres muqueuses ne trouvait pas d’anomalie. L’enfant a bénéficié d’un transit du grêle qui a objectivé des images correspondant à des polypes au niveau de l’intestin grêle dont la taille était inférieure à 10mm, et une fibroscopie digestive qui était normale.

L’examen de la fratrie a objectivé des cas similaires: le frère âgé de 7 ans, ayant un antécédent de méningite et une anophtalmie traumatique. Avec à l’examen cutané des lentigines au niveau du dos du nez évoluant depuis 1an, alors que l’examen des muqueuses était sans anomalie. La sœur, âgée de 5 ans, sans antécédents pathologiques notables, qui présentait depuis un an des lésions similaires avec à l’examen, des lentigines au niveau de la joue droite, au niveau de la lèvre inférieure et au niveau de la muqueuse buccale. Le transit du grêle et la fibroscopie digestive haute ont été réalisé chez les 2 enfants étaient normaux.

Une surveillance annuelle a été préconisée, mais les 3 enfants ont été perdus de vue.

### Consentement

Les auteurs déclarent avoir eu le consentement écrit des parents des trois enfants.

## Discussion

Le SPJ est une maladie rare de transmission autosomique dominante, défini par l’association d’une atteinte cutanée à type de lentiginose péri-orificielle (nez, lèvres, régions anale et génitale), d’une atteinte digestive, pulmonaire et des organes reproducteurs. En 1921, Jan Peutz, un médecin néerlandais a rapporté le cas d’une famille avec polypose gastro-intestinale et une lentiginose cutanéo-muqueuse [1]. En 1949, un médecin américain nommé Harold Joseph Jeghers a publié une description détaillée des patients présentant une polypose intestinale et une pigmentation de la peau, ce qui a conduit à l′identification du syndrome portant le nom des deux médecins [1,2,3]. La prévalence est estimé à 1 / 200 000, sans prédominance de sexe ou de race, l’âge moyen du diagnostic est de 22 ans sur une revue de 75 cas [4].

Le gène de PJS a été localisé sur le chromosome 19p13.13. Thakur et al ont affirmé l’existence de plus de 140 mutations différentes sur un gène supresseur de tumeur, nommé serine thréonine-kinase11 (STK11) codant pour une protéine la LKB1. Mc Garrity et Amos [4] ont noté que la LKB1 régule la voie de l’apoptose médiée par la p53, et joue un rôle dans le développement des tumeurs chez ces patients [1,5]. D’autres études ont suggéré l′existence d’un deuxième locus sur le même gène 19q13.4 identifié dans une minorité de familles [6], l’étude génétique n’a pas était réalisée chez nos enfants.

Les signes cutanés sont affichants et constituent un signe révélateur de la maladie mais ils ne sont pas les premiers à apparaitre. Dans tous les cas, ils sont à type de lentigines, ce sont des macules de couleur brune ou jaune chamois, irrégulièrement ovales et mesurent généralement moins de 5mm de diamètre. Ils siègent le plus souvent sur les lèvres, la muqueuse buccale, comme c’était le cas chez les 2 filles. La muqueuse nasale, les régions péri-orbitaire, les coudes, la face dorsale des doigts, les orteils et les régions plamo-plantaires peuvent également être touchés. Sur une revue de la littérature de 70 cas, les signes cutanés apparaissaient plus précocement chez les garçons (5-10ans) que chez les filles (10–15 ans), ce qui n’était pas le cas chez nos 3 trois enfants [7]. Les polypes digestifs constituent le deuxième signe cardinal de ce syndrome et ils peuvent révéler la maladie lorsqu’ils se manifestent d’emblée par leur complication telles les hémorragies digestives et les obstructions. Ces polypes sont des hamartomes qui se situent au niveau du jéjuno-iléon (90% des cas), du côlon (9% des cas) ou de l’estomac (24% des cas). Il n’y a pas de relation entre la taille des polypes et le potentiel dégénératif et on estime que 2/75 polypes peuvent dégénérer [8]. Les formes pédiatriques ne présentent pas de particularité clinique par rapport aux formes survenant chez l’adulte [7].

Les personnes atteintes de SPJ courent un risque accru de cancers, le risque cumulatif de cancer est estimé à 93%, Les organes les plus fréquemment touchés sont le tractus gastro-intestinal (oesophage, estomac, intestin grêle, côlon, rectum et pancréas) [3], le poumon [9], la prostate, le sein [10] et les organes reproducteurs [3,11]. Ces tumeurs peuvent être observées aussi bien chez les enfants que chez les adultes, avec une prévalence qui semble moindre chez les enfants [2].

La prise en charge des SPJ est basée principalement sur la surveillance et le traitement des polypes hamartomateux. La fréquence de surveillance des patients atteints de maladie de Peutz-Jeghers ne fait l’objet d’aucun consensus, mais il est souhaitable que les patients asymptomatiques bénéficient d’une endoscopie haute tous les 2 ans pour la surveillance et l′ablation des polypes. L’imagerie par résonance magnétique a montré un succès en tant que modalité de surveillance de l′intestin grêle et des testicules, l’échographie abdominale pour est réalisé pour le dépistage du cancer du pancréas. Chez les femmes, la mammographie, le test de Papanicolaou et l’échographie transvaginale sont effectués tous les 1 à 2 ans. Une numération-formule sanguine doit être réalisé pour détecter une anémie causée par les pertes sanguines [3,8]. Les épisodes occlusifs secondaire aux polypes sont spontanément résolutifs chez les enfants ainsi la polypectomie n’est pas systématique. Il n′existe aucun traitement standarisé pour la pigmentation cutanéo-muqueuse qui est présente chez la majorité des patients. Le traitement fait appel à la cryochirurgie, la dermabrasion et au laser Q-switched [3]. Vu les moyens économiques limités de nos enfants, seules des crèmes dépigmentantes ont été proposées.

## Conclusion

Le SPJ est une affection rare, les manifestations cutanées constituent le premier signe d’appel permettant ainsi un bilan à la recherche de néoplasie associée. Une surveillance étroite de ces malades est nécessaire à fin de détecter des cancers associé qui détermine ainsi le pronostic de ces malades.
